# If We Could Know

**Published:** 1891-08

**Authors:** Myra Wentworth Emerson


### IF WE COULD KNOW.

If we could know that we should sometime meet
  Our loved and lost upon some fairer shore,
Though bruised and bleeding were these weary feet,
  Though chill above our heads the black clouds lower.

Could we not patient bear life's seeming ill?
  The dreary path that must be short at best—
A little while, ah! yes, until, until
  We too shall reach that land of perfect rest!

If we could know that dear ones linger near
  To guide and cheer along the lonely way,
Would not the thought the saddest bosom cheer,
  And change the blackest night to perfect day?

If we could know—and yet it must be so;
  The soul within dies not with mortal breath;
"*If we could know,*" say not—for we *do know*
  In all this changeful world there is no death.

—MYRA WENTWORTH EMERSON, *Banner of Light.*

